# Our thanks to *Cilia’s* reviewers

**DOI:** 10.1186/2046-2530-2-4

**Published:** 2013-03-11

**Authors:** Phil Beales, Peter K Jackson

**Affiliations:** 1 UCL Institute of Child Health, London, WC1N 1EH, UK; 2 Genentech Inc., South San Francisco, CA, 94080, USA

## Abstract

**Contributing reviewers:**

We would like to thank all our reviewers for their contribution to the success of *Cilia.* Their voluntary participation has led to the launch of a new journal consistently publishing high quality articles and an intelligent peer review process for all our authors.

## 

Wolfgang Baehr

United States of America

Alexandre Benmerah

France

Joseph Besharse

United States of America

Daniel Carr

United States of America

Tamara Caspary

United States of America

Soren Christensen

Denmark

Dan Doherty

United States of America

Jonathan Eggenschwiler

United States of America

Guowei Fang

United States of America

Jacek Gaertig

United States of America

Raymond E Goldstein

United Kingdom

Ursula Goodenough

United States of America

Anna-Katerina Hadjantonakis

United States of America

Claire Jackson

United Kingdom

Dagan Jenkins

United Kingdom

Jian Jiao

China

Hannie Kremer

Netherlands

Tiansen Li

United States of America

Karel Liem

United States of America

Ben Margolis

United States of America

Jeffrey Martens

United States of America

John W McAvoy

Australia

Kimberly McDermott

United States of America

Hannah Mitchison

United Kingdom

Neil Morgan

United Kingdom

Roland Nitschke

Germany

Albert Ong

United Kingdom

Lotte Pedersen

Denmark

Ruman Rahman

United Kingdom

Sandra Rovira Amigo

Spain

Peter Satir

United States of America

Peter Schraml

Switzerland

Amelia Shoemark

United Kingdom

Deepti Trivedi

United States of America

John Wallingford

United States of America

Angus Wann

United Kingdom

Bradley Yoder

United States of America

